# Ecology and Spatial Distribution of Magnetotactic Bacteria in Araguaia River Floodplain

**DOI:** 10.1111/1758-2229.70073

**Published:** 2025-02-09

**Authors:** Igor Taveira, Jefferson Cypriano, Juliana Guimarães, Ludgero Cardoso Galli Vieira, José Francisco Gonçalves Junior, Alex Enrich‐Prast, Fernanda Abreu

**Affiliations:** ^1^ Instituto de Microbiologia Paulo de Góes Universidade Federal Do Rio de Janeiro Rio de Janeiro RJ Brazil; ^2^ Faculdade UnB Planaltina Universidade de Brasília Brasília DF Brazil; ^3^ Instituto de Ciências Biológicas Universidade de Brasília Brasília DF Brazil; ^4^ Multiuser Unit of Environmental Analysis Universidade Federal do Rio de Janeiro Rio de Janeiro RJ Brazil; ^5^ Department of Thematic Studies – Environmental Change Linköping University Linköping Sweden

**Keywords:** Araguaia River, biomineralization, magnetotactic bacteria, magnetotaxis

## Abstract

Magnetotactic bacteria (MTB) are Gram‐negative, ubiquitous, aquatic, flagellated, microaerophilic, or anaerobic microorganisms exhibiting magnetotactic behaviour based on magnetosomes, which are the structural signature of the group. Magnetosomes are ferrimagnetic nanocrystals surrounded by a lipid bilayer, usually aligned in chain(s) within the cell. Environmental abiotic conditions such as salinity, dissolved oxygen, pH, and oxidation–reduction potential may drive the diversity of MTB populations in environments. Our results reported the first evidence of MTB in sediments sampled from the Araguaia River floodplain in the Amazon‐Cerrado biome. Light microscopy showed at least six morphotypes of South‐seeking MTB. Transmission electron microscopy and energy‐dispersive X‐ray spectroscopy observations demonstrated magnetite cuboctahedral, prismatic, and anisotropic magnetosomes. PCA ordination demonstrated a more significant influence of depth, ORP (oxidation–reduction potential), and transparency in sampled data from the river main channel (MC). Non‐metric multidimensional scaling (NMDS) ordination and correlation analysis demonstrated a difference between MTB populations inhabiting MC and lakes and affluent (LA). NGS and bioinformatic analysis revealed higher richness and diversity among magnetotactic cocci and the majority phylogenetic assignment of MTB affiliated to Pseudomonadota phylum. Hence, the complete acquisition of these results will provide further insight into magnetotaxis characterisation and the abiotic factors that impact MTB spatial distribution.

## Graphical abstract

Magnetotactic bacteria, with their unique intracellular magnetic nanocrystals and magnetic field orientation, are widespread in aquatic environments. However, the discovery of these bacteria in the Araguaia River, a part of two crucial biomes in South America, the Cerrado and the Amazon, marks a significant breakthrough. Our research revealed a rich diversity of this group in the river's courses. Ilha do Bananal, the largest fluvial island in the world, emerged as a hotspot for these microorganisms.

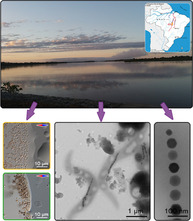

## 1 Introduction

Magnetotactic bacteria (MTB) are ubiquitous Gram‐negative prokaryotes that are capable of biomineralizing magnetosomes, ferrimagnetic nanocrystals enveloped by a lipid bilayer, which aid the cell's orientation along geomagnetic field lines while navigating in chemically stratified water columns (Bazylinski and Frankel 2004). The passive alignment with the geomagnetic field combined with active flagellar propulsion is called magnetotaxis. Magnetotaxis is an efficient mechanism assisting chemotaxis in a unidirectional movement to find optimal conditions to survive and grow, maximising proton‐motive force (Frankel, Williams, and Bazylinski 2006). The biomineralization of magnetosomes is highly controlled through a set of genes organised in clusters within the bacterial genome (known as magnetosome gene clusters, MGCs) (Uebe and Schüler 2016; Lin et al. 2018).

In their majority, MTB are mesophilic and present in aqueous environments harbouring pH proximal to neutrality (Lin, Pan, and Bazylinski 2017; Lin, Paterson et al. 2017). Exceptions are the moderately halophilic multicellular magnetotactic prokaryotes (MMPs) (Abreu et al. 2007), thermophilic (Lefèvre et al. 2010), obligately alkaliphilic (Lefèvre et al. 2011b), psychrophilic (Abreu et al. 2016), and acidophilic MTB (Abreu et al. 2018). Hence, it is proposed that different natural environments shape specific MTB populations according to physical–chemical parameters, imposing different selective pressures. Salinity is among the most impactful factors driving MTB population diversity (Simmons et al. 2004). In eutrophic lakes with high organic matter content, the coccoid and ovoid morphotypes are predominant, while in oligotrophic waters, MTB populations comprise higher diversity (Flies, Peplies, and Schüler 2005). It is discussed that magnetotactic cocci are the most abundant in diversified aqueous environments (Lin et al. 2014). Although the magnetotactic behaviour is dispersed in different phyla from the Bacteria domain, its origin and scatter remain unknown.

The Araguaia River, in the Araguaia‐Tocantins basin, represents one of the most important rivers in the central region of Brazil. It is the largest hydrographic basin located entirely in Brazilian territory. The basin comprehends the states of Tocantins, Goiás, Mato Grosso, Pará, Maranhão, and the Federal District (Valente, Latrubesse, and Ferreira 2013). The river course is divided into three regions: high, medium, and low Araguaia. The medium Araguaia course outlines the confluence of important affluents (e.g., Crixás, Mortes, and Cristalino rivers). The medium Araguaia houses a floodplain with numerous lakes and affluents, which contribute to the maintenance of the diversity and the proper functioning of ecosystems in the region (Latrubesse e Steuvax, Latrubesse and Steuvax 2006) Considering this scenario, extensive floodplains have been dismantled.

Meanwhile, agricultural lands experienced considerable expansion in addition to large urbanised areas (Brinson and Malvárez 2002). In medium Araguaia, there was a modification in twelve lakes for land use, mainly in the states of Goiás and Mato Grosso. Uttermost lakes located in Tocantins state are best preserved by accounting for (i) areas close to indigenous preservation reserves and (ii) Bananal island conservation unity (Garcia et al. 2017). Additionally, Tocantins state claims the lowest deforestation rate in the region (Sano et al. 2010). The Araguaia River runs through two unique biomes, Cerrado and Amazônia, known for higher diversity. Thus, the study of this site will contribute to a better comprehension of MTB ecology and diversity, comprising an area located in a tropical floodplain.

## 2 Experimental Procedures

### 2.1 Sampling Sites and Magnetic Enrichment

Sediment and water samples were collected along the main channel of the Araguaia River and its affluents and lakes (Table S1) using cylindrical opaque plastic bottles (radius = 10 cm; height = 20 cm). Samples were harvested within the sediment–water interface. Sampling was performed in January 2019. Abiotic factors such as pH, temperature (°C), oxidation–reduction potential (ORP) (mV), electrical conductivity (mS/cm), total dissolved solids in water (TDS) (g/L), turbidity (NTU), dissolved oxygen (DO) (mg/L and %) and transparency (cm) were acquired during the sampling using a U‐50 series multi‐parameter water quality checker (HORIBA, Kyoto, Japan). Depth of sediments was assessed with a laser measuring tape (BOSCH, Stuttgart, Germany). Sediments were transported at room temperature and were stored and protected from light. MTB were enriched by placing a cylinder small neodymium magnet (diameter = 2 mm; height = 1 mm) outside the bottle for 15 min. MTB were collected and washed with local water previously filtered in 0.22 μm membrane.

### 2.2 Light Microscopy

Magnetically enriched MTB were observed by hanging drop technique Wenter et al. (2009) in a differential interference contrast (DIC) microscope (AxioImager D2, ZEISS, Oberkochen, Germany) coupled with acquisition cameras (AxioCam HRm and AxioCam MRc). Magnetotactic behaviour was evaluated by providing an external magnetic field generated by a small neodymium magnet placed on the microscope's stage. MTB quantification, in each sampled site, was performed based on previously acquired images by enriching cells with magnetotactic behaviour in the edge drop. In triplicates, five images per sampled site were used for MTB quantification at five different image fields in a similar way performed for TEM‐processed images described below. Posteriorly, mean and standard deviation were calculated based on acquired images.

### 2.3 Transmission Electron Microscopy (TEM) and Energy Dispersive X‐Ray Spectroscopy (EDS)

For direct observation under a TEM, magnetically enriched cells were deposited on top of formvar‐coated 300 mesh copper grids and dried with filter paper. Cells were imaged using a FEI Morgagni 268 TEM (FEI, Eindhoven, Netherlands) operated at 80 kV, a Tescan VEGA 3 LMU SEM (Brno‐Kohoutovice, Czech Republic) equipped with an X‐Max 20 mm^2^ SDD (Oxford Instruments, Abingdon, UK) or a JEOL 2100F Cold FEG (JEOL, Tokyo, Japan) operated at 200 kV equipped with a Xplore 80 mm^2^ SDD (Oxford Instruments, Abingdon, UK). The software AZtec (Oxford Instruments, Abingdon, UK) was used for the EDS mapping and point analysis. Length, width, and shape factor were measured using the iTEM software suite (Olympus, Tokyo, Japan). Magnetosome morphology count was performed by magnetically enriching MTB in an edge drop on top of a formvar‐coated 300 mesh copper grids. Grids were posteriorly dried with filter paper. Three replicates were chosen for each sampled site, and manual count was performed using 5 different image fields per grid (Figure S1). One‐way ANOVA was performed to infer statistical differences in shape factor. Multiple comparisons between groups were performed using Tukey's multiple comparison test. For the magnetosomes dimensions, a linear regression model was fitted with R^2^ values calculated using GraphPad Prism 8.0 (GraphPad Software, California, EUA).

### 2.4 Abiotic Factor Correlation With MTB morphotypes' Diversity and Magnetosome Morphologies

All samples were tested for normality using the Shapiro‐Wilker test (GraphPad Software, California, USA). Principal component analysis (PCA) ordination was performed for abiotic factors using Past 4.0 and data were treated using the *z*‐score function (Hammer, Harper, and Ryan 2001). Groups were distinguished by samples collected in the river main channel (MC) and lakes and affluents (LA).

Non‐metric multidimensional scaling (NMDS) was performed for data of MTB quantification and magnetosome diversity in sampled sites using Past 4.0. Groups were distinguished by samples collected in the river MC and LA. Abiotic factors were input as environmental variables to comprehend the influence of those over MTB morphology variation along Araguaia River floodplain. Euclidean distance matrix was chosen, with outputs of stress value. A one‐way PERMANOVA was performed to infer statistical differences between the two groups in PCA and NMDS data using Past 4.0 as well as Pearson correlation matrix.

### 2.5 16S rRNA Gene Next‐Generation Sequencing (NGS), bioinformatic Analysis, and Alpha‐ and Beta‐Diversity Metrics

Magnetically enriched samples were purified using a capillary race‐track technique (Wolfe, Thauer, and Pfennig 1987), in triplicates, for sampling sites P11 (LA) and P02 (MC). DNA was extracted using the GeneJET Genomic DNA Purification Kit (REF: K0721; Thermo‐Fisher, USA) according to manufacturers' protocol promptly after samples were collected. Whole genomic DNA amplification was performed using a REPLI‐g Cell WGA kit (REF: 150052; Qiagen, USA). Nucleic acid purity and concentration were assessed using a NanoDrop 1000 device (Thermo‐Fisher, USA) and sequenced at NGS Soluções Genômicas (Piracicaba, Brazil) on MiSeq equipment using paired‐end runs (2 × 250) according to the manufacturer's guidelines. Primers used were 515F (GTG YCA GCM GCC GCG GTA A) (Walters et al. 2016) and 926R (CCG YCA ATT YMT TTR AGT TT) (Quince et al. 2011), which target the V3‐V4 hypervariable region of the 16S rRNA gene.

Bioinformatic analyses were performed using the mothur software version 1.44.3 (Schloss et al. 2009). Alpha‐ (i.e., richness, diversity, dominance and evenness) and beta‐diversity metrics (OTU relative abundance and distribution) were exported from software for later processing and plotting. Complete bioinformatics pipeline and statistical analysis are provided within supplementary information.

Sequence data were deposited in the NCBI Sequence Read Archive (SRA) and are available under BioProject accession numbers PRJNA1025865.

## 3 Results

### 3.1 Sampling Sites, PCA and Correlation of Abiotic Factors

**FIGURE 1:**
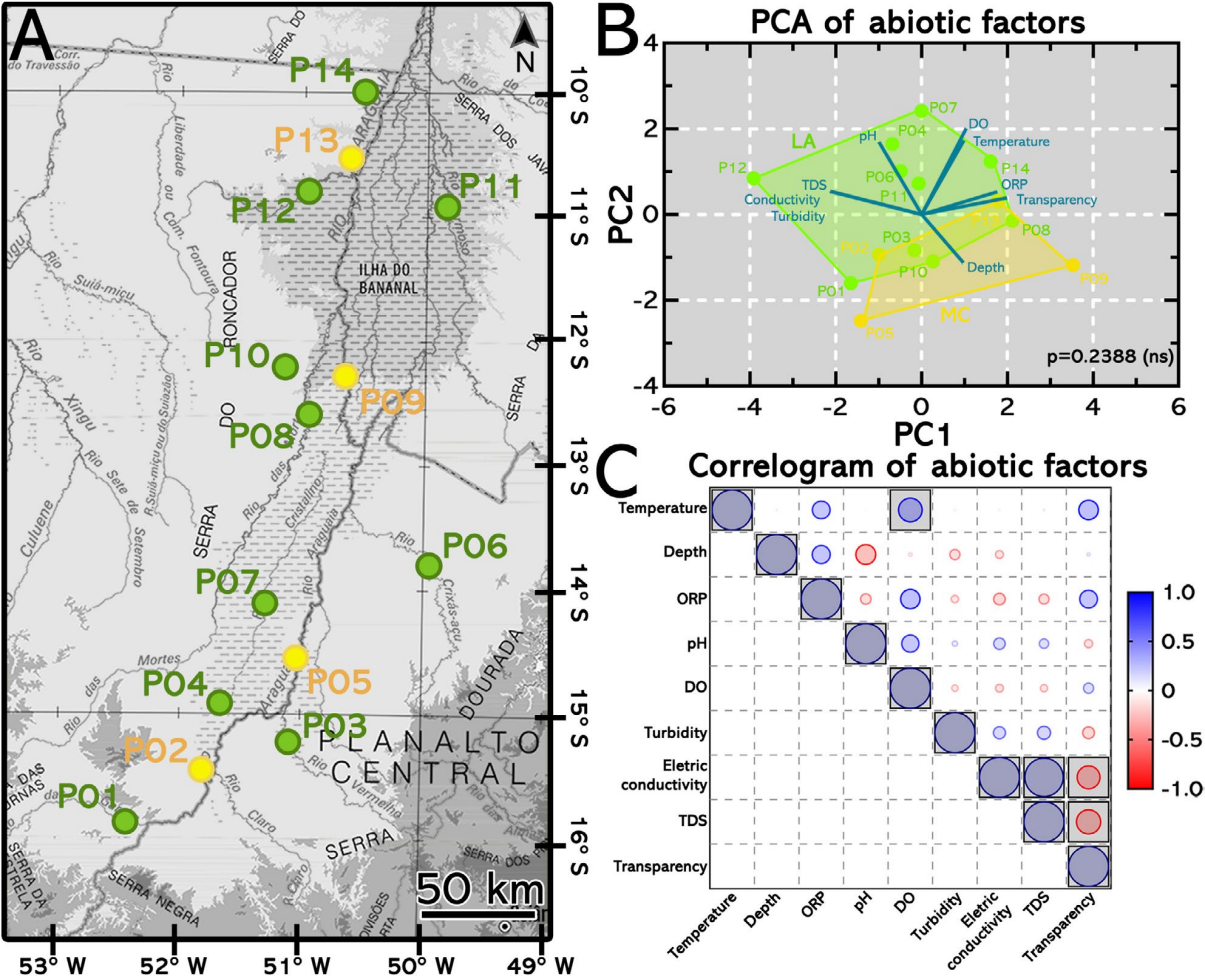
Sampling sites in the Araguaia River floodplain with its lakes and affluents and principal component analysis of abiotic factors. (A) Geographic localization of sampling sites which were divided in: (i) sampling sites in river main channel (MC; yellow); and (ii) sampling sites in lakes and affluents (LA, green). (B) PCA of abiotic factors. PC1 (38.09%) and PC2 (21.68%) combined are responsible for accommodating 59.77% of data variance. No statistical differentiation between the clusters of sampling sites from MC and LA were found (ns, *p* = 0.2388). Sampling sites from MC had variance in their abiotic factors better explained by components ORP, transparency and depth, while sampling sites from LA encompassed all abiotic factors components in its cluster. (C) Correlogram of abiotic factors variation along the Araguaia floodplain. Boxed values represent statistically significant correlations (*p* < 0.05). Strong positive and statistically significant correlations were found among: (i) temperature and DO (mg/L) (Pearson *r* = 0.5578); and (ii) electric conductivity (mS/cm) and TDS (Pearson *r* = 0.9563). Strong negative and statistically significant correlations were found among: (i) transparency and TDS (Pearson *r* = −0.6027); and (ii) transparency and electric conductivity (Pearson *r* = −0.5700).

The water–sediment interface was sampled for 14 different sites across the Araguaia River floodplain (Figure 1A). The data set encompasses approximately 900 km of the Araguaia River. Sites were divided into MC and LA depending on the sampling site. The abiotic factors measurements of each sampling site are displayed in the Table S1. Together, components PC1 (38.09%) and PC2 (21.68%) hold 59.77% of the variance present in the data. No statistical group differentiation was observed (*p* = 0.2388) (Figure 1B). ORP, transparency, and depth better explain variance in the abiotic factors of MC sampling sites. LA sampling sites encompassed all abiotic factors' components in their cluster. The correlation between the abiotic factors is displayed in Figure 1C. Strong positive and statistically significant correlations were found among (i) temperature and DO (mg/L) (Pearson r = 0.5578); and (ii) electric conductivity (mS/cm) and TDS (Pearson r = 0.9563). Strong negative and statistically significant correlations were found among (i) transparency and TDS (Pearson r = −0.6027) and (ii) transparency and electric conductivity (Pearson r = −0.5700).

### 3.2 Magnetotactic Response, MTB Morphotypes and Its Distribution Along the Araguaia River Main Channel and Its Affluents

**FIGURE 2:**
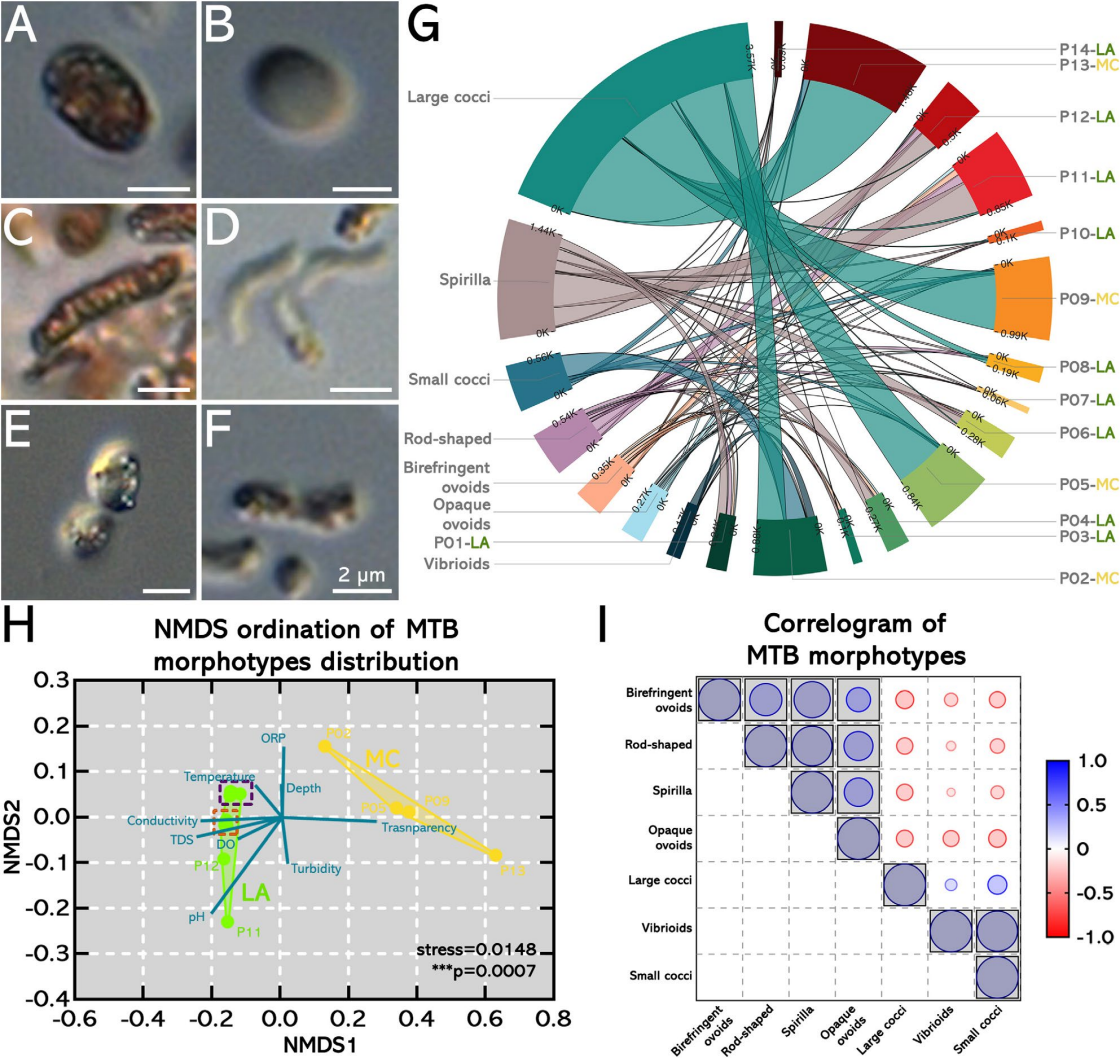
MTB morphotype and its distribution along the Araguaia River floodplain. (A–F) Magnetically enriched MTB observed in DIC microscopy. MTB were classified in six morphotypes: (i) birefringent ovoid (A); (ii) opaque ovoid (B); (iii) rod‐shaped (C); (iv) spirillum (D); (v) large cocci (E); and (vi) vibrioid (F). Small cocci are not represented in DIC images. (G) Chord plot of MTB morphotypes distribution regarding each sampling site. Coccoid MTB (both large and small) prevailed in the river MC whilst rod‐shaped bacteria, ovoids (both birefringent and opaque), and spirilla prevailed in LA. Vibrioid cells were found in both MC and LA. Large cocci accounted for almost half of all morphotype diversity identified in Araguaia River floodplain. (H) NMDS ordination of MTB morphotypes distribution. MTB morphotypes detected in river MC did not display intersections with morphotypes detected in LA. Evidence is supported by statistical inference which separates both groups (***p = 0.0007) presenting good data accommodation (stress = 0.0148). The environmental variables ORP, depth and water transparency exerted greater influence over MTB sampled in MC whilst temperature, DO, pH, TDS and electric conductivity exerted greater influence over MTB sampled in LA. Three narrow clusters were identified, as: (i) P05 and P09 (MC); (ii) P03, P07, P08, P10, and P14 (LA); and (iii) P01, P04, and P06 (LA). (I) Correlogram of MTB morphotypes. Boxed values represent statistically significant correlations (*p* < 0.05). Correlogram supports group differentiation observed in NMDS ordination (H). Strong positive and statistically significant correlations were found among vibrioid cells, large and small cocci which were the predominant morphotypes in MC. Strong positive and statistically significant correlations were found among rod‐shaped bacteria, ovoids (both birefringent and opaque) and spirilla which were the predominant morphotypes in LA.

Based on DIC microscopy observations, MTB morphotypes were classified as: (i) birefringent ovoid (Figure 2A), which contains birefringent inclusions in the cell's cytoplasm; (ii) opaque ovoid (Figure 2B); (iii) rods (Figure 2C); (iv) spirilla (Figure 2D); (v) large cocci (Figure 2E); and (vi) vibrioid cells (Figure 2F) (Videos S1 and S2). Small cocci (smaller than 1 μm) were not demonstrated in DIC images due to low image resolution; yet were quantified the same way for each sampling site. Large cocci (approximately 1 μm) were the most abundant morphotype (51.73%). Large and small cocci were primarily in the MC, whereas rods, both ovoids, spirilla, and vibrioid cells were predominantly in LA (Figure 2G).

Difference between MTB morphotypes in river MC and LA samples was observed (***p = 0.0007; stress = 0.0148). Higher values of ORP, water transparency, and depth components majorly influenced MTB morphotypes identified in MC. In LA samples, MTB were significantly influenced by higher temperature, electric conductivity, pH, and turbidity, DO, and STD components (Figure 2 H). The highest number of magnetotactic spirilla and rods were found in lakes P11 (pH 6.7) and P12 (pH 6.6), where the pH was higher compared to other sites, being proximal to neutrality, as both sites belong to the Bananal island plain. The highest number of birefringent ovoids were observed in the two clusters formed by lake sites (dashed lines), where components DO, temperature, conductivity and TDS exert greater influence. LA site P01 was the only anoxic watercourse in which no cocci and vibrioid cells were observed. Nevertheless, the two MC sites P05 and P02 were among the locations harbouring low DO values. In addition, MC sites P05 and P02 evinced the lowest abundances of magnetotactic cocci among other MC sites. Turbidity, depth, and transparency allegedly exerted minor influence on MTB morphotype spatial distribution. Endorsed on Pearson correlation matrix, performed to support NMDS data, it was possible to infer: (i) strong positive and statistically significant correlations (*p* < 0.05) were found among vibrioid cells, large and small cocci which were the predominant morphotypes in MC; (ii) strong positive and statistically significant correlations (p < 0.05) were found among rod‐shaped bacteria, ovoids (both birefringent and opaque) and spirilla which were the predominant morphotypes in LA (Figure 2H; Table S2).

**FIGURE 3:**
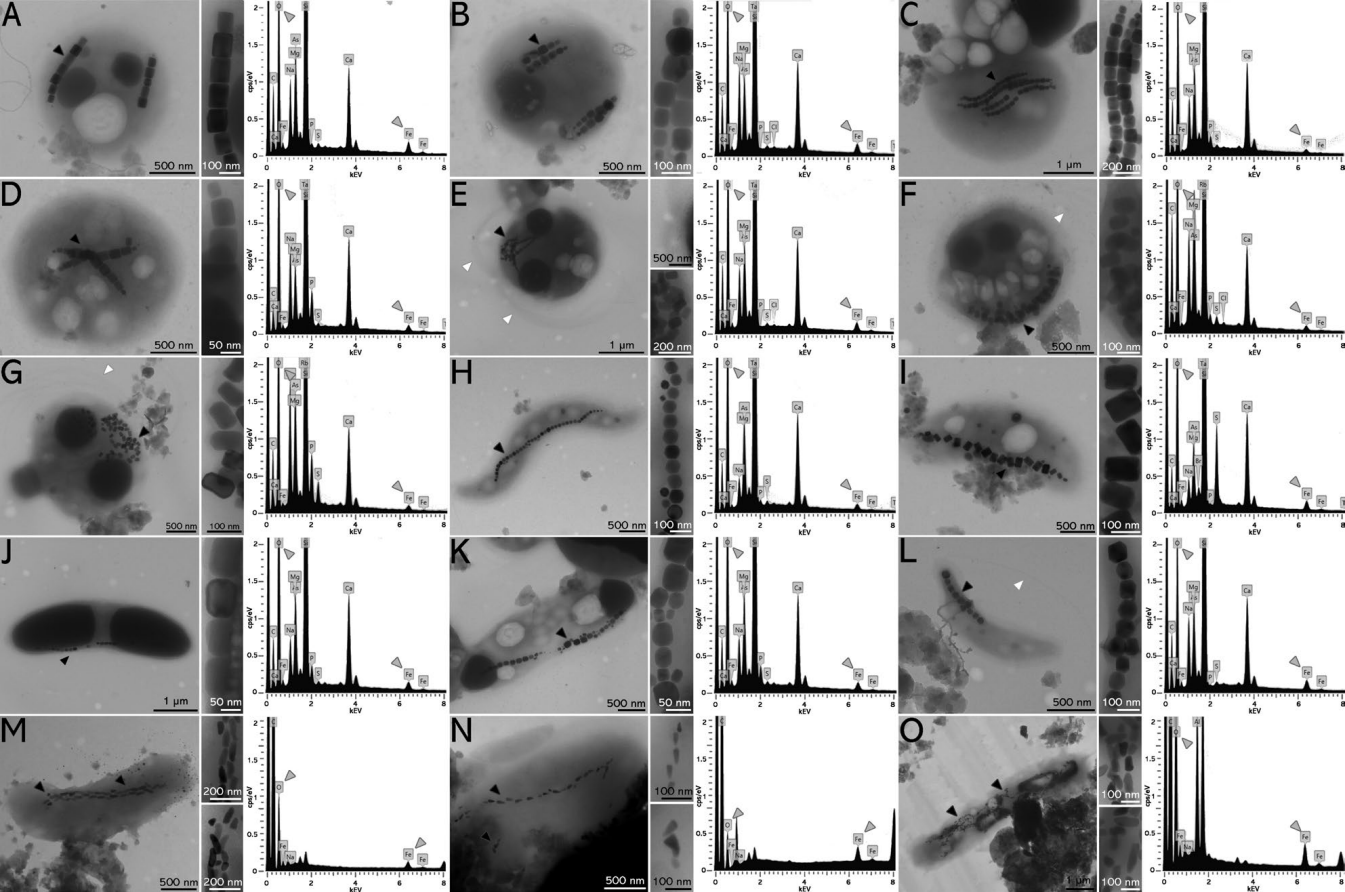
Magnetically‐enriched MTB observed by transmission electron microscopy (TEM) and energy‐dispersive X‐ray spectroscopy (EDS) microanalysis of magnetosome. From left to right: TEM image of MTB, respective magnetosome chain in higher magnification inset, and EDS spectrum of selected region in magnetosome chain (black arrowhead). Flagella indicated by white arrowheads. (A–G) Coccoid and coccoid‐ovoid MTB with: (i) two parallel magnetosome chains (A); (ii) double parallel magnetosome chains (B); (iii) multiple magnetosomes apparently organised in chains parallel to each other (C); (iv) two magnetosome chains (D); (v) scattered magnetosomes seem to be near flagella bundle insertion (E); (vi) aggregated magnetosomes seem to be aligned with cell edge (F); (vii) dispersed magnetosomes seem to be proximal to flagella bundle (G). (H) Spirillum with cuboctahedral magnetosomes aligned to cell's major axis and electron‐lucent inclusions. (I–L) Vibrioid MTB with magnetosomes aligned to cell's major axis and some cells containing polar electron‐dense inclusions. (M–O) Rod‐shaped MTB with anisotropic magnetosomes. (M, N) Rods with anisotropic magnetosome single‐chain aligned with cell's major axis. (O) Rod with multiple anisotropic magnetosomes apparently aligned to cell's major axis. All EDS spectra comprised oxygen and iron peaks (grey arrowheads), indicating iron oxide magnetosomes, possibly magnetite.

Magnetically enriched MTB in sampled sites were observed in TEM (Figure 3). Flagella bundles attached to a cell's body could be observed (Figure 3; white arrowheads). In the MC samples, the following MTB morphotypes were observed: (i) coccus with two or more parallel chains of octahedral magnetosomes containing electron‐lucent and electron‐dense inclusions (Figure 3A–D); and (ii) coccus with octahedral magnetosomes, apparently not organised in parallel chains, containing electron‐dense and electron‐lucent inclusions with flagellum in one's cell pole (Figure 3E). In LA samples reported magnetotactic cells were: (i) ovoid cells containing prismatic magnetosomes not organised in chains as well as the presence of two electron‐dense granules in cell's extremities and a flagella bundle (Figure 3F,G); (ii) spirillum with a single chain of cuboctahedral magnetosomes aligned with the cell's major axis (Figure 3H); (iii) vibrio with a single chain of prismatic magnetosomes aligned with the cell's major axis (Figure 3I); (iv) vibrio with a single chain of magnetosomes aligned with the cell's major axis and presenting electron‐dense inclusions at the poles of the cell (Figure 3J); (v) vibrio with a single chain of magnetosomes aligned with the cell's major axis and presenting electron‐dense and electron‐lucent granules (Figure 3K); (vi) vibrio with a single chain of cuboctahedral magnetosomes aligned with the cell's major axis (Figure 3L); and (vii) rods with multiple chains of anisotropic magnetosomes aligned with the cell's major axis (Figure 3M–O). EDS spectra of selected magnetosomes displayed the characteristic Fe‐Kα and O‐Kα peaks and the absence of S‐Kα, indicating that all were composed of iron oxides. However, as some samples present minor S‐Kα signal we performed the same analysis acquiring spectra from the cell's cytoplasm and outside the cell to indicate that S‐Kα peaks were provided by other inclusions or belonged to the environment (Figure 3).

### 3.3 Diversity and Distribution of Magnetosomes Along the Araguaia River Floodplain

**FIGURE 4:**
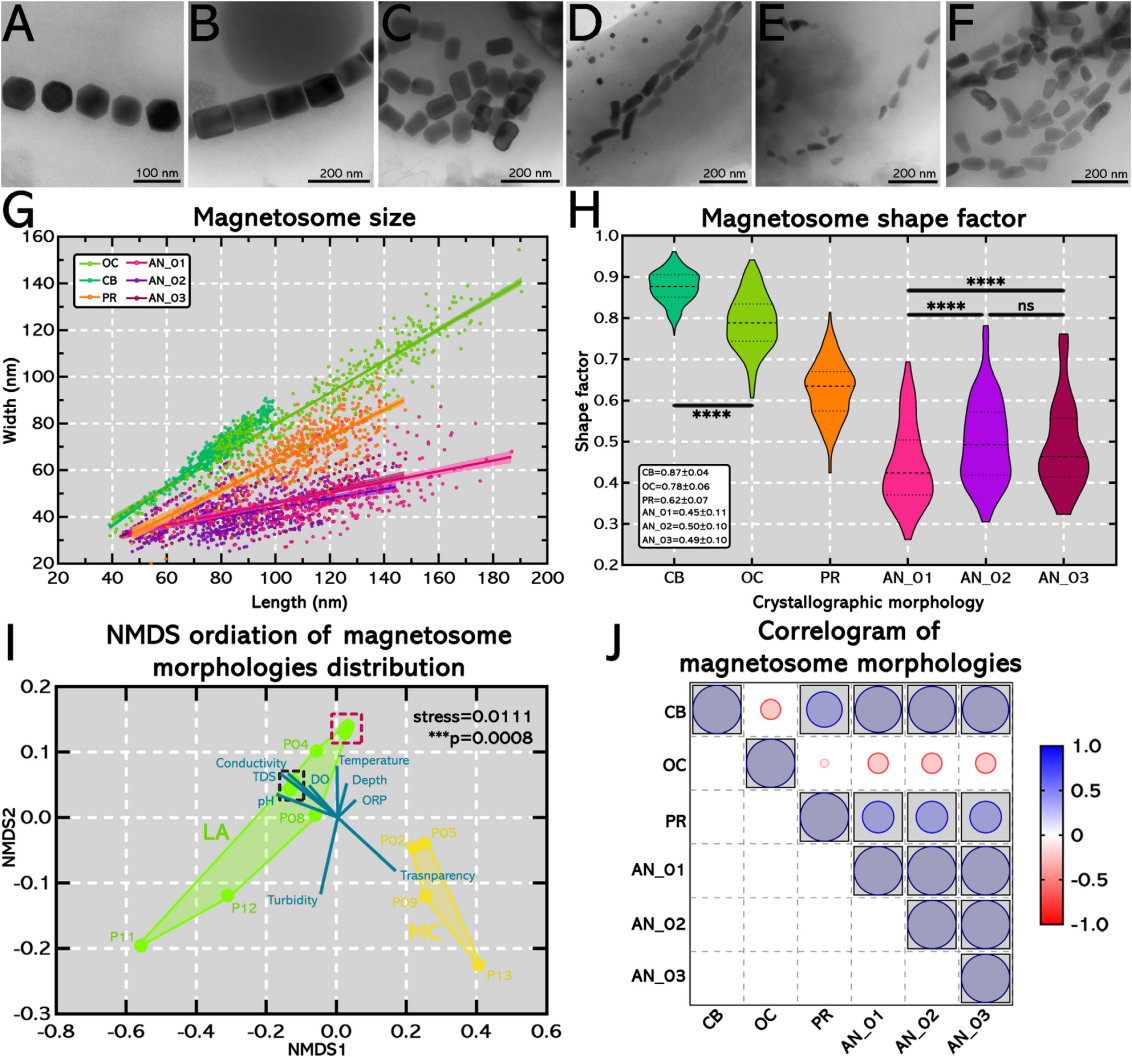
Magnetosome diversity, dimensions, shape factor and its distribution along Araguaia river floodplain. (A–F) TEM images of magnetosome diversity found in MTB in MC and LA. (A) Cuboctahedral magnetosomes. (B) Octahedral magnetosomes. (C) Prismatic magnetosomes. (D–F) Anisotropic magnetosomes. Some rod‐shaped cells had a thicker cell body, which hinders the visualisation and assessment of magnetosomes dimensions. (G) Dispersion plot of magnetosomes dimensions (length vs. width). Linear regression model was fitted and 95% confidence index is plotted. Legend: (i) cuboctahedral magnetosomes (CB; cyan); (ii) octahedral magnetosomes (OC; green); (iii) prismatic magnetosomes (PR; orange); and (iv) anisotropic magnetosomes (AN_01, AN_02, AN_03; light pink, purple and magenta, respectively). (H) Violin plot of magnetosome shape factor distribution. There is statistical significance between: (i) cuboctahedral and octahedral magnetosomes (****p < 0.0001); (ii) anisotropic 01 and 02 (****p < 0.0001); and (iii) anisotropic 01 and 03 (****p < 0.0001). (I) NMDS ordination of magnetosome morphology distribution. Magnetosomes identified in river MC (yellow) were clustered and exhibited no intersection with magnetosomes identified in LA (green). Evidence is supported by statistical inference which separates both groups (***p = 0.0008) presenting good data accommodation (stress = 0.0111). All environmental variables, except water transparency, exert greater influence over magnetosome diversity in LA. Three narrow clusters were observed regarding magnetosomes morphological distribution: (i) sampling sites P02 and P05 in MC group; (ii) sampling sites P03, P07, P10 and P14 of LA group; and (iii) sampling sites P01 and P06 of LA group. (J) Correlogram of magnetosome diversity. Boxed values represent statistically significant correlations (p < 0.05). Correlogram supports group differentiation observed in NMDS ordination (I). Strong positive and statistically significant correlations were found among cuboctahedral, prismatic and the three different anisotropic magnetosomes, which are predominant in LA. Octahedral magnetosomes, predominant in MC, exhibited negative correlation with all other magnetosome morphologies observed.

Magnetosomes observed in TEM were sorted into: (i) cuboctahedra (CB) (Figure 4A); (ii) octahedra (OC) (Figure 4B); (iii) prismatic (PR) (Figure 4C); and (iv) three different sizes of anisotropic crystals (AN_01, AN_02, AN_03) (Figure 4D–F, respectively). Linear regression model of magnetosomes dimensions returned: (i) a good fitting for CB magnetosomes, strictly associated with magnetotactic spirilla, and OC magnetosomes, strictly associated with magnetotactic cocci; (ii) satisfactory fitting for PR magnetosomes, strictly associated with magnetotactic ovoids; and (iii) poor fittings regarding the three AN magnetosomes, strictly associated with magnetotactic rods (Figure 4G) (Table S3). Shape factor of CB was significantly different (*p* < 0.0001) from shape factor of OC magnetosomes. Anisotropic magnetosomes had the lowest shape factor values, and statistical significance was inferred between: (i) AN_01 and AN_02 (p < 0.0001); and (ii) AN_01 and AN_03 (p < 0.0001) (Figure 4H).

Good data accommodation was achieved (stress = 0.0111) and there was statistical difference between magnetosomes catalogued in LA and MC (***p = 0.0008) (Figure 4I,J). Magnetosomes of MTB inhabiting LA were majorly influenced by all abiotic factors' components, except water transparency. The highest number of cuboctahedral, prismatic and anisotropic magnetosomes (AN_01, 02 and 03) were found in the LA sites P11 (pH 6.7) and P12 (6.6) where pH is higher compared to other sites, being proximal to neutrality. Those sites are located in the Bananal island which possesses: (i) distinct values of ORP (ranging 119 mV) and DO (ranging 2.6 mg/L or 37.2%); but (ii) similar values of temperature (ranging 0.3°C). NMDS data was endorsed on Pearson correlation matrix (Figure 4K). Correlation indexes and p‐values are displayed in Table S4.

### 3.4 Alpha‐ and Beta‐Diversity Metrics and Phylogenetic Affiliation of Magnetically Enriched Bacteria

**FIGURE 5:**
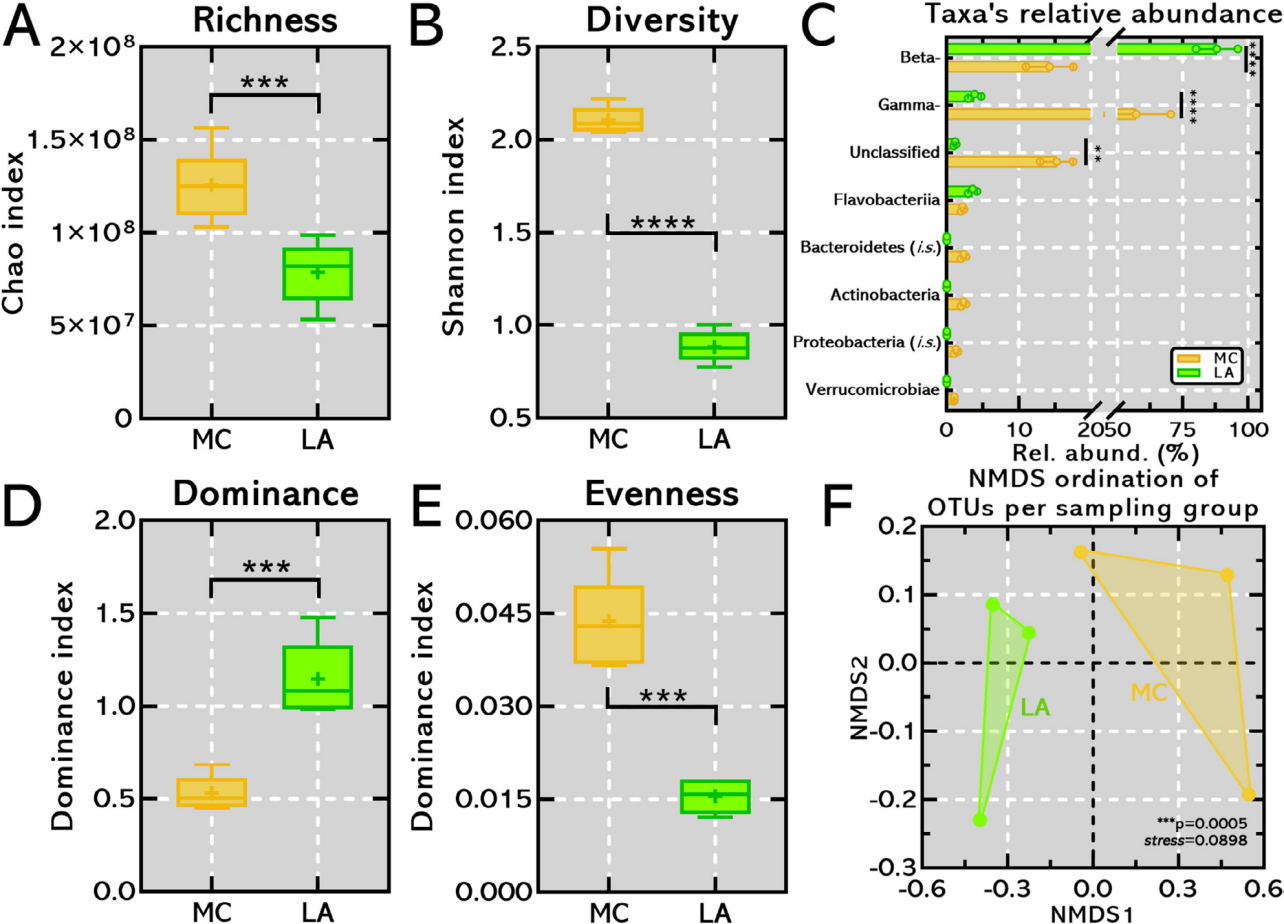
Alpha‐ and beta‐diversity metrics of two MTB hotspots from LA and MC in magnetically‐enriched sample. (A, B, D, E) Alpha diversity metrics. MTB species richness (*p* = 0.001), diversity (p < 0.0001) and evenness (*p* = 0.0001) are statistically significant higher within magnetically‐enriched bacteria from river MC while comprising lower dominance index (*p* = 0.0003). (C) Relative abundance of OTUs affiliated to Betaproteobacteria class are statistically significant higher in MC compared to LA (p < 0.0001) whereas OTUs affiliated to Gammaproteobacteria class are statistically significant higher in LA compared to MC (p < 0.0001). (F) NMDS ordination of OTUs per sampling group demonstrated no statistically significant change between microbial community structure. Good data accommodation was achieved with stress = 0.0898 with statistics' inference showing difference between MC and LA clusters (*p* = 0.0005).

Regarding alpha‐diversity metrics, OTUs retrieved from MC displayed higher species richness (1.25 ± 0.18 × 10^8^) and diversity (2.10 ± 0.07) with statistical difference compared to LA (***p = 0.0010 and < 0.0001, respectively) (Figure 5). Major components of magnetotactic microbial community were OTUs affiliated with: (i) Betaproteobactetia class predominantly in LA (88% ± 8%) than MC (14.3% ± 3.3%); (ii) Gammaproteobacteria class predominantly in MC (57.2% ± 13.2%) than LA (3.9% ± 0.9%); and (iii) taxonomic unassigned OTUs predominantly in MC (15.3% ± 2.3%) (Figure 5C). Regarding beta‐diversity, magnetotactic microbial community structure was also different comparing MTB clusters from MC and LA, which are displayed in NMDBs with good data fitting (*p = 0.0005; stress = 0.0898) (Figure 5F), corroborating previous observations (Figures 2 and 3).

## 4 Discussion

### 4.1 Abiotic Factor Variation Along the Araguaia River Floodplain

Abiotic factor values acquired during the sampling were dimensionally reduced to highlight their variation along the Araguaia River main channel and its lakes and affluents. The two groups (MC and LA) manifested an overlapping region in PCA analysis (*p* = 0.2388). Sampling sites from MC were often clustered in regions close to LA sites. Nonetheless, no locations were overlapped after PCA, indicating that despite some similarities in their abiotic factors, they consist of unique spots with particular characteristics (Figure 1B). Maximum depth observed was 7.3 m (Table S1). The lakes of the Araguaia River floodplain were shallow, as described previously. Maximum depth varied depending on river seasonality, which ranges from (i) 0.3 m to 4.55 m during the low stage and (ii) 2.7 to 7.75 during the high stage (Morais et al. 2005). Electric conductivity did not exhibit significant fluctuations as the river's main channel and its tributaries are composed of freshwater (Nabout, Nogueira, and Oliveira 2006).

Thirteen of fourteen sites exhibited pH lower than 7.0, ranging from 5.22 to 6.70 (Table S1). Moderate oxygen saturation and temperature observed in this study were typical of tropical environments such as medium Araguaia River floodplain (Alves et al. 2019). This positive correlation between temperature and DO is commonly observed in tropical oligotrophic floodplains (Ford, Boon, and Lee 2002). In this example, DO exhibited a moderate positive and not statistically significant correlation with ORP and pH (Figure 1C). These findings follow prior observations in the medium Araguaia (Machado et al. 2016; Alves et al. 2019). The observed pH values were weakly acidic, consistent with previous reports (Johnson and Hallberg 2003). Soil acidity in Cerrado, a tropical savanna, is mainly caused by (i) leaching of bases from the soil profile, (ii) use of ammoniacal fertilisers, (iii) by proton generation in the soil, (iv) mineralization of organic matter, (v) intensive crop cultivation; and (vi) N_2_ fixation by legumes/bacteria symbionts (Fageria and Nascente 2014). In this particular case, land use is one factor that contributes most to weakly acidic waters (Alves et al. 2019). The predominance of low pH values may be expected in tropical floodplain lagoons, considering these systems usually have a low to moderate ORP (de Carvalho et al. 2001). ORP, in this situation, was moderately positive. However, there was no statistically significant correlation with DO, temperature, and depth (Figure 1C). The lower ORP values indicate decaying matter in the water that cannot be cleared or decompose (Horne and Goldman 1994), which are usually registered next to urban centres and agricultural farms (e.g., sites P05 and P06 close to Luis Alves city).

Interestingly, the MC site P09 (Figure 1A) exhibited the lowest turbidity value and the second highest transparency value (Table S1). Land use for agriculture and urbanisation are important factors driving the alterations in water transparency (Alves et al. 2019). For instance, lakes are expected to exhibit greater transparency in the dry season than in the rainy season. This results from a lower amount of suspended solids (TDS) and turbidity during the dry season, which is usually enhanced by water inflow during the flood (Thomaz, Bini, and Bozelli 2007). In this study, samples were collected during the rainy season, which can explain the elevated turbidity values caused by the constant resuspension of riverine sediment (de Oliveira Marcionilio et al. 2016). Transparency exhibited a strong negative and statistically significant correlation with TDS and electric conductivity (Figure 1C). Despite its correlation with electric conductivity, it was demonstrated that electric conductivity has a minor effect with a narrow range of fluctuation (Table S1). The medium Araguaia River consists of a freshwater floodplain with weakly acidic, low transparency, moderate oxygen saturation, low to moderate ORP, and shallow waters.

### 4.2 MTB Characterisation, Phylogenetic Affiliation and Their Spatial Distribution

Advances in studies of metacommunities, the process responsible for explaining microbial biogeography, have been achieved in the last few years. However, they are still not fully understood (Chen et al. 2008; Lindström and Langenheder 2012). A prevalent thought was that microbial distribution was only determined by the environmental characteristics regarding microbial cell small size, high abundance, and high dispersal rates (Fenchel and Finlay 2004). This principle is in concordance with Baas Becking (Baas‐Becking 1934), which asserts “everything is everywhere, but the environment selects”. However, spatial dispersal limitation may occur in microorganisms (Martiny et al. 2006). Environmental components of the aquatic biosphere, such as pH (Gong et al. 2015), conductivity (Simon et al. 2015), phosphorus concentration (Triadó‐Margarit and Casamayor 2012), luminosity (Charvet, Vincent, and Lovejoy 2014), primary productivity (Simon et al. 2015), temperature, and depth (Gong et al. 2015; Wang et al. 2015), can promote shifts in microbial community composition. Spatial patterns of communities can be induced by intrinsic factors of the organism (e.g., competition, predation, migration rate, dispersion capacity). This promotes an autocorrelation in data or extrinsic factors (interaction with other spatially structured factors, such as environmental characteristics) that generate spatial dependence (Sokal and Oden 1978; Legendre & Fotin, Legendre and Fortin 1989).

Current knowledge of abiotic factors influencing MTB population dynamics relies majorly on ellipsoid MMPs (eMMPs) and spherical MMPs (sMMPs) (Simmons et al. 2004; Martins et al. 2012; Du et al. 2015; Liu et al. 2018). MMPs have been strictly reported inhabiting non‐freshwater environments (Lin et al. 2014). Geochemical analysis at the brackish Lake Yehu, China, revealed that the sMMPs can tolerate seasonal changes better than eMMPs, which prefer more stable conditions. Optimal temperature is the most influencing abiotic factor in the MMP population in Lake Yehu. Despite that, other factors, including salinity, sulphate, TOC, and TDS, probably provoke shifts in sMMPs abundance (Du et al. 2015). When comparing the vertical profile of ORP in Lake Yehu, China, with its seasonal shifts, it was possible to infer that a high abundance of MMPs was associated with high temperatures and low ORP. These findings suggest high MMP abundances are favoured in an environment with active organic catabolism. In addition, high concentrations of ammonium and silicate were associated with low abundances of both eMMPs and sMMPs (Liu et al. 2018). MMP abundance variations were observed in a Brazilian hypersaline lagoon (Martins et al. 2009; Martins et al. 2012) and a salt pond in the USA (Simmons et al. 2004; Simmons, Bazylinski, and Edwards 2007). No MMP was reported in the Araguaia River floodplain, suggesting once more that they are strictly associated with saline waters (Figure 2). These results from MTB are consistent with the observation that salinity is one of the primary drivers of global distribution in bacterial diversity (Lozupone and Knight 2007). Besides salinity, additional environmental factors could shape MTB population dynamics like (i) temperature (Lin, Wang, and Pan 2012), (ii) ORP (Simmons, Bazylinski, and Edwards 2006; Lin et al. 2013), (iii) light (Shapiro et al. 2011), (iv) redox state of sulphur compounds (Postec et al. 2012), and (v) iron bioavailability (Lin et al. 2013).

Thus, this is the first description of MTB in the Araguaia River and accounts for the first MTB report in a tropical freshwater floodplain. MTB in the Araguaia River exhibited a South‐seeking behaviour following current literature (Lin, Pan, and Bazylinski 2017; Lin et al. 2017). Coccoid and vibrioid morphotypes were prevalent in MC along with their respective magnetosome morphologies (i.e., octahedral and prismatic), where they are mainly related to low values of DO, TDS, and pH and higher transparency values, suggesting these MTB possibly prefer acid, more microaerophile to anoxic and transparent water conditions' when compared with MTB from LA. On the other hand, rods, spirilla, and ovoids, dominant in LA, seem to tolerate higher DO and pH values (Figure 2). So far, magnetotactic rods have been found among freshwater or low‐salinity ecosystems (Lefèvre et al. 2010; Jogler et al. 2010) and at low tide zones of marine environments such as the Yellow Sea (Teng et al. 2018). The same distribution pattern was reported by magnetotactic spirilla from the *Magnetospirillum* genus (Matsunaga, Sakaguchi, and Tadakoro 1991). The same distribution pattern was compiled by magnetotactic spirilla from the *Magnetospirillum* genus (Matsunaga, Sakaguchi, and Tadakoro 1991). Magnetotactic spirilla and rods along with their respective magnetosome morphologies (i.e., cuboctahedral, curved anisotropic and bullet‐shaped), observed in the medium Araguaia floodplain, corroborate these observations as the river is a freshwater watercourse with near to zero electric conductivity (lesser than 0.04 mS/cm) (Table S1). Magnetotactic vibrios do not exhibit the same restriction, in which they are found in (i) mud slurries from freshwater sediments (Kolinko et al. 2013) and (ii) sulphide‐rich sediments from salt marsh (Bazylinski, Frankel, and Jannasch 1988). In addition to that, magnetotactic vibrios along with their magnetosomes' morphologies were mainly dispersed in the river MC (Figures 1 and 2). Together, small and large cocci account for nearly 60% of the relative abundance of magnetosome morphotypes found in the Araguaia River (Figure 2). It is discussed that magnetotactic cocci are the most abundant in various environments (Lin et al. 2014; Liu et al. 2021). These cocci exhibit high diversity in terms of size, morphology, and magnetosome organisation (Spring et al. 1993). Their distribution is consistent with descriptions previously made for freshwater MTB species (Liu et al. 2021). For instance, higher species richness was observed in the P12 and P12 sites (i.e., most turbid waters), which could be explained as some MTB when exposed to light, reversed their magnetotactic behaviour to become NS, as they tend to prefer more turbid and less transparent water columns (Shapiro et al. 2011).

Magnetotactic rods are generally affiliated with the phyla Nitrospirota and, Desulfobacterota, (Jogler et al. 2010; Lefèvre et al. 2011a; Lin, Pan, and Bazylinski 2017; Lin et al. 2017) and Gammaproteobacteria class (Lefèvre et al. 2012; Li et al. 2017; Liu et al. 2022). These rod‐shaped organisms predominantly possess magnetosomes that vary in shape depending on their taxonomic affiliation. Nitrospirota synthesise curved bullet‐shaped magnetosomes, while Desulfobacterota produce straight bullet‐shaped magnetosomes. These magnetosomes are typically composed of magnetite, organised into multiple chains aligned with the longer cell axis. Additionally, various cytoplasmic inclusions contribute to birefringence, which were observable under DIC microscopy (Figure 2, Figure S2) (Jogler et al. 2010; Li, Liu, et al. 2020; Li, Menguy, et al. 2020). Magnetotactic ovoids, reported in LA samples, are generally affiliated with the phylum Nitrospirota in literature (Figure 3). All ovoids affiliated with the Nitrospirota phylum described in the literature possess anisotropic curved magnetosomes composed of magnetite, organised into multiple chains aligned with the longer cell axis (Kolinko et al. 2013; Qian et al. 2019).

Freshwater magnetotactic spirilla have only been described within the Alphaproteobacteria class, characterised by cuboctahedral magnetosomes composed of magnetite (Matsunaga, Sakaguchi, and Tadakoro 1991; Dziuba et al. 2016). Spirilla were also observed using conventional TEM, containing a single chain of cuboctahedral magnetite magnetosomes aligned with the longer cell axis (Figure 3H). Magnetotactic vibrios have, so far, been found in species affiliated within Beta‐ (Abreu et al. 2018) and Alphaproteobacteria classes (Bazylinski and Frankel 2004) and the Nitrospirota phylum (Lefèvre et al. 2010). At least four different vibrioid cell types were observed (Figure 3I to L). All these magnetotactic vibrios possess a single chain of prismatic, cuboctahedral, or undefined‐morphology magnetite magnetosomes aligned with the longer cell axis (Figure 3K). Some strains have electron‐dense inclusions at the cell ends (Figure 3J,K). Interestingly, OTUs affiliated to Betaproteobacteria class were detected in MC and LA samples (Figure 5C), which could possibly indicate an acidophilic vibrio with similar characteristics to the literature, as previously described (Abreu et al. 2018).

Magnetotactic cocci, commonly associated with the class Alphaproteobacteria within the Pseudomonadota phylum, have been a subject of extensive research (Bazylinski et al. 2013; Morillo et al. 2014; Lin et al. 2018; Liu et al. 2021). The candidate class Etaproteobacteria has been proposed to reclassify some magnetotactic cocci based on genomic evidence (Ji et al. 2017), although it has yet to be officially recognised in international databases. This study did not recover any OTUs affiliated with either the Alphaproteobacteria class or the candidate class Etaproteobacteria in the MC samples, based on sequencing and analysis of the V3‐V4 hypervariable region of the 16S rRNA gene. The use of WGA might have introduced amplification bias, potentially affecting the representation of certain microbial taxa in the analysis performed in this study. Nevertheless, the unique characteristics of this biome and the challenging access to the region make the ecological data and spatial distribution shown in the study crucial to understanding the global distribution of MTB. The absence of identification of a phylogenetic group of magnetotactic cocci might indicate that the partial analysis of the 16S coding gene and thresholds used might not have the appropriate resolution for OTU classification in this case, potentially affecting the classification of magnetotactic cocci. Possibly, due to low abundance of those sequences in databases, miss affiliation in other classes (e.g., Alpha‐) or could be assigned to unclassified sequences, which comprise 15.3% ± 2.3% of MC (Figure 5). Different cocci morphologies were observed using conventional TEM (Figure 3). For instance, MTB within the phylum Pseudomonadota are known to synthesise magnetosomes with octahedral, cuboctahedral, or prismatic morphologies. This morphological variation is closely linked to the evolutionary adaptations of MTB, reflecting their phylogenetic affiliations and environmental niches (Li, Liu, et al. 2020; Li, Menguy, et al. 2020).

### 4.3 Magnetosome Characterisation and Their Spatial Distribution

The classification of magnetosomes' morphologies presented in this study acknowledges the inherent limitations of the imaging techniques, particularly the absence of refined methods (e.g., electron tomography), which could provide more accurate structural characterisation. However, important limitations that hindered the usage of those techniques were the remote location and challenging accessibility of the Araguaia River floodplain, as well as the difficulty in processing high‐quality samples for advanced TEM analyses (Morais et al. 2005; Nabout, Nogueira, and Oliveira 2006). Nevertheless, our classification aligns with findings from previous studies, which report similar morphological variations in magnetotactic bacteria, reinforcing the ecological and taxonomic significance of our observations (Lins and Farina 2004; Lin et al. 2013; Liu et al. 2021; Amor et al. 2022).

Additionally, the NMDS revealed two distinct groups of magnetosome morphologies in MC and LA with statistical difference (****p‐value = 0.0008). Thus, all abiotic factor components could exert some influence on the diversity of magnetosomes found in LA and there are few reports in the literature about the influence of various environmental factors on magnetosome populations. The linear model was satisfactory for accommodating magnetosome width and length data for CB, OC, PR (Figure 4; Figure S2). Anisotropic curved crystals were associated with rods from the Nitrospirota phylum and did not fit size prediction models. This may be related to a more lenient genetic control of magnetosome biomineralization since this is the ancestral phylum of MTB described so far. The divergence between the Pseudomonadota and Nitrospirota phyla likely occurred during the Archean eon approximately 2.7 billion years ago (Lin, Pan, and Bazylinski 2017; Lin et al. 2017). In contrast, cuboctahedral, octahedral, and prismatic magnetosomes have a narrower and well‐defined size range, fitting linear prediction models. The shape and size of magnetosomes biomineralized by MTB in these classes could be controlled by more recent and precise genetic mechanisms in the evolution of MTB. Additionally, the real morphological diversity of anisotropic magnetosomes' has only been revealed recently, describing their possible three stage growth, starting as an isotropic crystalline habit then migrating to anisotropy (Li et al. 2015; Li, Menguy, et al. 2020; Li, Liu, et al. 2020). Thus, this comprises a difficult asset in fitting anisotropic magnetosomes' models, as authors suggest that they must be indexed and zone axis identified before proper assignment.

## 5 Conclusion

This is the first report of MTB in a tropical floodplain from the Cerrado‐Amazon biome, the Araguaia River floodplain, located in Brazilian territory. In the Araguaia River floodplain, MTB populations and their magnetosomes exhibited a spatial distribution that could be influenced by abiotic factors that distinguish the populations present in MC and LA. For instance, magnetotactic cocci and vibrios along with their respective magnetosome morphologies (i.e., octahedral and prismatic), abundant in MC, could be driven by higher values of ORP, water transparency, and depth and lower values of temperature, pH, turbidity, DO, and STD components. Magnetotactic spirilla and rods along with their respective magnetosome morphologies (i.e., cuboctahedral, curved anisotropic and bullet‐shaped) were more abundant in Bananal Island, the world's largest fluvial island, with pH values more proximal to neutrality than at other sampling sites. Additionally, although magnetotactic cocci is considered a single MTB morphology, they exhibited higher diversity within MTB community evaluated by sequencing a hypervariable fragment of the *rrs* gene.

Spatial distribution was also observed within magnetosomes' morphological signature, despite the limitations in determining magnetosome shape based on conventional TEM images and the challenges of sample accessibility, as anisotropic, cuboctahedral, and prismatic magnetosomes were mainly located in LA and possibly driven by higher temperature, electric conductivity, pH, turbidity, DO, and STD components. Besides morphological characterisation, 16S rRNA gene sequencing based on the V3‐V4 hypervariable region from the magnetically enriched samples affiliated most OTUs to Beta‐ and Gammaproteobacteria class from the Pseudomonadota phylum. Alpha‐ and beta‐diversity metrics corroborated the spatial distribution observed within MTB from MC and LA and demonstrated a higher species diversity and richness among magnetotactic cocci. Hence, the abiotic factors analysed in the context of flooding season in the medium Araguaia River dictate MTB spatial distribution as was inferred by morphological and molecular analysis.

## Author Contributions

**Igor Taveira:** conceptualization, investigation, writing – original draft, methodology, writing – review and editing. **Jefferson Cypriano:** investigation, writing – original draft, writing – review and editing, methodology. **Juliana Guimarães:** investigation, writing – original draft, methodology, writing – review and editing. **Ludgero Cardoso Galli Vieira:** methodology, writing – original draft, writing – review and editing. **José Francisco Gonçalves Junior:** funding acquisition, project administration, writing – original draft, writing – review and editing. **Alex Enrich‐Prast:** funding acquisition, writing – original draft, writing – review and editing, conceptualization. **Fernanda Abreu:** conceptualization, writing – original draft, writing – review and editing, supervision, funding acquisition, methodology.

## Conflicts of Interest

The authors declare no conflicts of interest.

## Supporting information


**Data S1** Supporting Information.

**Video S1.** Magnetotactic response of magnetically enriched samples from Araguaia floodplain’s lakes and affluents observed in DIC microscopy. Rods, spirilla, vibrioid and ovoid (both opaque and birefringent) cells are the majorly identified MTB morphotypes in lakes and affluents. Compass, placed on the microscope’s stage, represents the external magnetic field orientation. Magnetic north is represented in red whereas magnetic south is represented in blue. MTB, globally, exhibit south‐seeking behaviour, swimming antiparallel to the external magnetic field.

**Video S2.** Magnetotactic response of magnetically enriched samples from Araguaia river main channel observed in DIC microscopy. Coccoid MTB, both large and small, are predominant in the river main channel. Compass, placed on the microscope’s stage, represents the external magnetic field orientation. Magnetic north is represented in red whereas magnetic south is represented in blue. MTB, globally, exhibit south‐seeking behaviour, swimming antiparallel to the external magnetic field.

## Data Availability

The data that support the findings of this study are openly available in NCBI Sequence Read Archive at https://pubmed.ncbi.nlm.nih.gov/, reference number PRJNA1025865.
