# Supplementary material for: Ecology and Spatial Distribution of Magnetotactic Bacteria in Araguaia River Floodplain
Source: Environ Microbiol Rep. 2025 Feb 9;17(1):e70073. doi: 10.1111/1758-2229.70073 (PMC11807442; doi:10.1111/1758-2229.70073)
Supplement: Supplementary file 1 — Data S1 Supporting Information. [file EMI4-17-e70073-s002.docx]

**Ecology and spatial distribution of magnetotactic bacteria encountered in Araguaia River floodplain**

Igor Taveira^1^, Jefferson Cypriano^1^, Juliana Guimarães^1^, Ludgero Cardoso Galli Vieira^2^, José Francisco Gonçalves Junior^3^, Alex Enrich-Prast^4,5^, Fernanda Abreu^1§^

# Supporting information

# 1. Supplementary tables and figures

**Supplementary table 1.** Sampling sites and abiotic factors acquired using a multi-parameter water quality checker

| **Sampling site** | **Condition** | **Date** | **Latitude (S)** | **Longitude (W)** | **Temperature (°C)** | **Depth (m)** | **ORP** | **pH** | **DO (%)** | **Turbidity** | **Conductivity (mS/cm)** | **TSD (g/L)** | **Transparency (cm)** |
| --- | --- | --- | --- | --- | --- | --- | --- | --- | --- | --- | --- | --- | --- |
| **P01** | **LA** | 19/01/2019 | 14º57'54.5" | 51º07'10.5" | 29,40 | 3,10 | 110,00 | 5,70 | 0,00 | 4,00 | 0,05 | 0,03 | 100,00 |
| **P02** | **MC** | 19/01/2019 | 14º43'49.3" | 51º02'09.9" | 28,50 | 3,40 | 283,00 | 5,60 | 38,10 | 5,70 | 0,05 | 0,03 | 78,00 |
| **P03** | **LA** | 18/01/2019 | 14º07'47.9" | 50º55'35.2" | 29,30 | 5,60 | 288,00 | 5,79 | 50,00 | 6,90 | 0,04 | 0,03 | 86,10 |
| **P04** | **MC** | 17/01/2019 | 13º46'08" | 50º52'38.8" | 28,90 | 0,80 | 216,00 | 7,30 | 83,60 | 3,50 | 0,04 | 0,02 | 117,50 |
| **P05** | **MC** | 16/01/2019 | 13º35'21.3" | 50º45'46.9" | 26,40 | 3,90 | 76,00 | 5,75 | 28,30 | 4,80 | 0,03 | 0,02 | 68,00 |
| **P06** | **LA** | 15/01/2019 | 13º22'44.3" | 50º40'41.6" | 31,75 | 2,80 | 198,00 | 6,10 | 73,80 | 5,40 | 0,04 | 0,03 | 106,00 |
| **P07** | **LA** | 21/01/2019 | 13º23'20.2" | 50º35'59.2" | 33,60 | 3,70 | 272,00 | 6,10 | 107,00 | 4,70 | 0,06 | 0,04 | 130,00 |
| **P08** | **LA** | 23/01/2019 | 12º38'53.7" | 50º42'21.7" | 30,60 | 3,60 | 318,00 | 5,41 | 88,50 | 6,20 | 0,02 | 0,01 | 100,70 |
| **P09** | **MC** | 23/01/2019 | 12º42'05.2" | 50º43'06.9" | 29,80 | 5,40 | 319,00 | 5,22 | 67,40 | 2,50 | 0,02 | 0,01 | 198,00 |
| **P10** | **LA** | 23/01/2019 | 12º36'45.6" | 50º40'11.5" | 29,70 | 7,30 | 290,00 | 5,85 | 42,40 | 6,00 | 0,04 | 0,03 | 107,00 |
| **P11** | **LA** | 24/01/2019 | 12º20'20.0" | 50º39'16.4" | 29,30 | 4,80 | 249,00 | 6,70 | 92,20 | 4,80 | 0,04 | 0,03 | 100,00 |
| **P12** | **MC** | 24/01/2019 | 11º48'55.0" | 50º41'47.8" | 29,00 | 1,90 | 143,00 | 6,60 | 55,00 | 14,20 | 0,06 | 0,04 | 49,00 |
| **P13** | **MC** | 24/01/2019 | 11º51'21.4" | 50º47'25.0" | 31,30 | 2,60 | 248,00 | 5,89 | 52,80 | 9,30 | 0,02 | 0,01 | 209,00 |
| **P14** | **LA** | 25/01/2019 | 11º32'10.6" | 50º45'26.7" | 31,08 | 3,90 | 237,00 | 6,53 | 108,90 | 4,10 | 0,02 | 0,01 | 130,00 |

**Legend:** LA: lakes and affluents; MC: main channel.

**Supplementary table 2.** Correlation matrix with Pearson r (r) and p-value (p) of MTB morphotypes diversity

| **-** | **Birefringent ovoids** | | **Opaque ovoids** | | **Rods** | | **Spirilla** | | **Large cocci** | | **Small cocci** | | **Vibrios** | |
| --- | --- | --- | --- | --- | --- | --- | --- | --- | --- | --- | --- | --- | --- | --- |
|  | **r** | **p** | **r** | **p** | **r** | **p** | **r** | **p** | **r** | **p** | **r** | **p** | **r** | **p** |
| **Birefringent ovoids** | 1,00 | 0,0000 | 0,58 | 0,0299 | 0,77 | 0,0012 | 0,87 | 0,0001 | -0,43 | 0,1262 | -0,38 | 0,1755 | -0,32 | 0,2681 |
| **Opaque ovoids** | 0,58 | 0,0299 | 1,00 | 0,0000 | 0,70 | 0,0057 | 0,68 | 0,0073 | -0,40 | 0,1571 | -0,42 | 0,1378 | -0,38 | 0,1780 |
| **Rods** | 0,77 | 0,0012 | 0,70 | 0,0057 | 1,00 | 0,0000 | 0,95 | 0,0000 | -0,39 | 0,1694 | -0,34 | 0,2282 | -0,22 | 0,4405 |
| **Spirilla** | 0,87 | 0,0001 | 0,68 | 0,0073 | 0,95 | 0,0000 | 1,00 | 0,0000 | -0,39 | 0,1665 | -0,31 | 0,2776 | -0,20 | 0,5013 |
| **Large cocci** | -0,43 | 0,1262 | -0,40 | 0,1571 | -0,39 | 0,1694 | -0,39 | 0,1665 | 1,00 | 0,0000 | 0,45 | 0,1023 | 0,28 | 0,3322 |
| **Small cocci** | -0,38 | 0,1755 | -0,42 | 0,1378 | -0,34 | 0,2282 | -0,31 | 0,2776 | 0,45 | 0,1023 | 1,00 | 0,0000 | 0,97 | 0,0000 |
| **Vibrios** | -0,32 | 0,2681 | -0,38 | 0,1780 | -0,22 | 0,4405 | -0,20 | 0,5013 | 0,28 | 0,3322 | 0,97 | 0,0000 | 1,00 | 0,0000 |

**Legend:** OC: octahedral magnetosomes; PR: prismatic magnetosomes; AN_01: anisotropic magnetosomes morphology 01; AN_02: anisotropic magnetosomes morphology 02; AN_03: anisotropic magnetosomes morphology 03; r: Pearson correlation coefficient; p: p-value.

**Supplementary table 3.** Linear regression of magnetosomes size

| **MTB** | **Magnetosome** | **Abbreviation** | **n** | **Linear regression** | **R^2^** |
| --- | --- | --- | --- | --- | --- |
| Spirillum | Cuboctahedral | CB | 421 | *w* = 0.67ℓ + 11.92 | 0.9091 |
| Coccus | Octahedral | OC | 526 | *w* = 0.83ℓ + 2.71 | 0.8849 |
| Ovoid | Prismatic | PR | 477 | *w* = 0.57ℓ + 5.65 | 0.6003 |
| Rod-shaped_01 | Anisotropic 01 | AN_01 | 475 | *w* = 0.22ℓ + 22.84 | 0.2922 |
| Rod-shaped_02 | Anisotropic 02 | AN_02 | 516 | *w* = 0.20ℓ + 23.59 | 0.2371 |
| Rod-shaped_03 | Anisotropic 03 | AN_03 | 246 | *w* = 0.24ℓ + 21.57 | 0.4825 |

**Legend:** *w= width;* ℓ = length.

**Supplementary table 4.** Correlation matrix with Pearson r (r) and p-value (p) of magnetosome diversity

| **OC** | | **PR** | | **AN_01** | | **AN_02** | | **AN_03** | |
| --- | --- | --- | --- | --- | --- | --- | --- | --- | --- |
| **r** | **p** | **r** | **p** | **r** | **p** | **r** | **p** | **r** | **p** |
| -0,41 | 0,1444 | 0,73 | 0,0029 | 0,95 | 0,0000 | 0,95 | 0,0000 | 0,95 | 0,0000 |
| 1,00 | 0,0000 | -0,16 | 0,5769 | -0,41 | 0,1402 | -0,41 | 0,1402 | -0,41 | 0,1402 |
| -0,16 | 0,5769 | 1,00 | 0,0000 | 0,65 | 0,0117 | 0,65 | 0,0117 | 0,65 | 0,0117 |
| -0,41 | 0,1402 | 0,65 | 0,0117 | 1,00 | 0,0000 | 1,00 | 0,0000 | 1,00 | 0,0000 |
| -0,41 | 0,1402 | 0,65 | 0,0117 | 1,00 | 0,0000 | 1,00 | 0,0000 | 1,00 | 0,0000 |
| -0,41 | 0,1402 | 0,65 | 0,0117 | 1,00 | 0,0000 | 1,00 | 0,0000 | 1,00 | 0,0000 |

**Legend:** OC: octahedral magnetosomes; PR: prismatic magnetosomes; AN_01: anisotropic magnetosomes morphology 01; AN_02: anisotropic magnetosomes morphology 02; AN_03: anisotropic magnetosomes morphology 03; r: Pearson correlation coefficient; p: p-value.

**
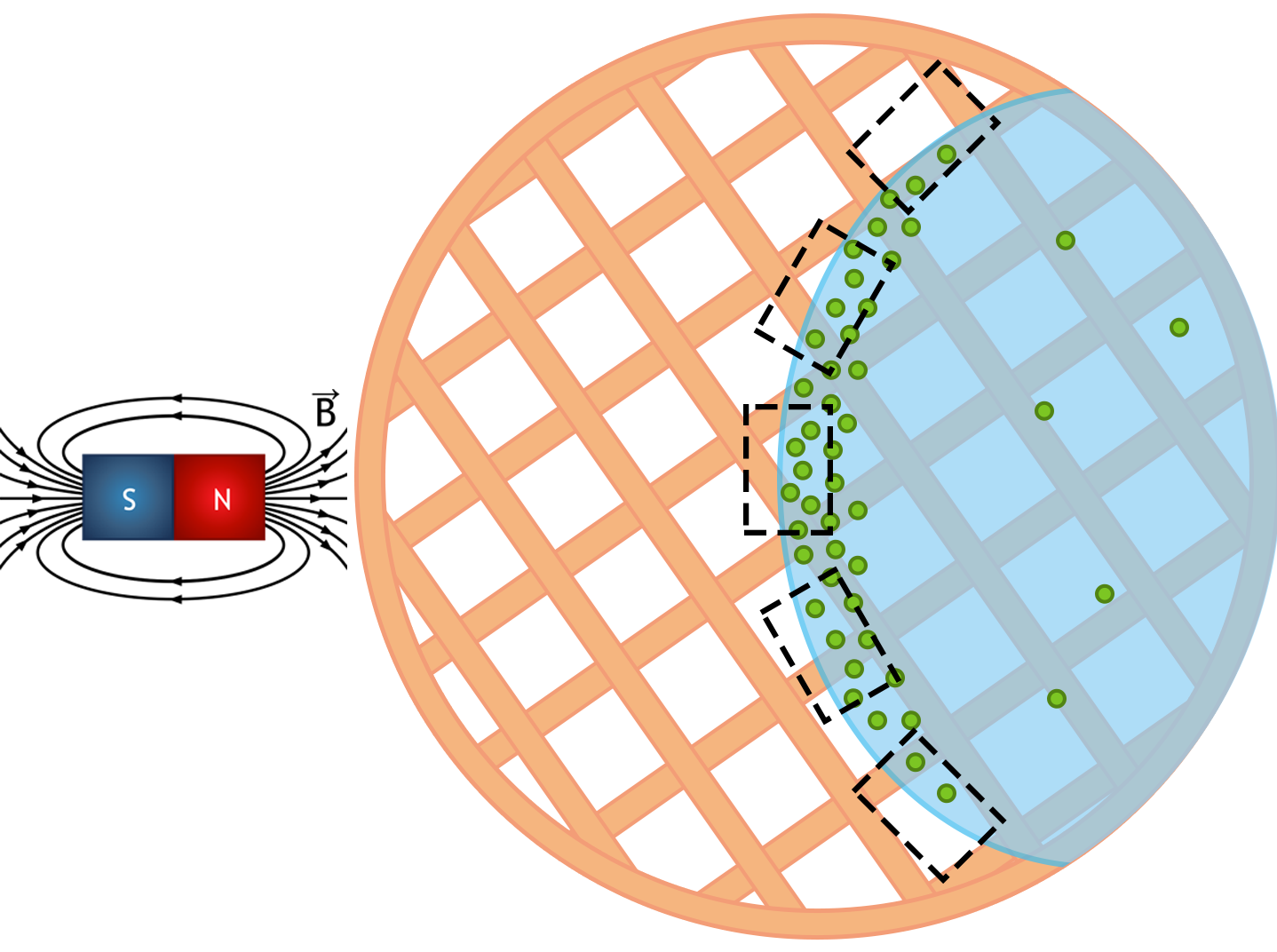
**

**Supplementary figure 1.** Magnetosome quantification protocol on TEM grid. A drop of magnetically enriched cells (green dots) is placed in a TEM grid in order to form a drop edge. Cells with a magnetotactic response swim antiparallel to the small magnet placed near the TEM grid, thus accumulating in the edge drop. The North magnetic pole is represented in red while the South magnetic pole is represented in blue. Five different fields (dashed rectangles) are selected to maximize the scanning area present in the edge drop. Three replicates for each sampling site are observed to obtain mean and standard deviation per site. Magnetosomes are quantified regarding their morphological diversity.

**
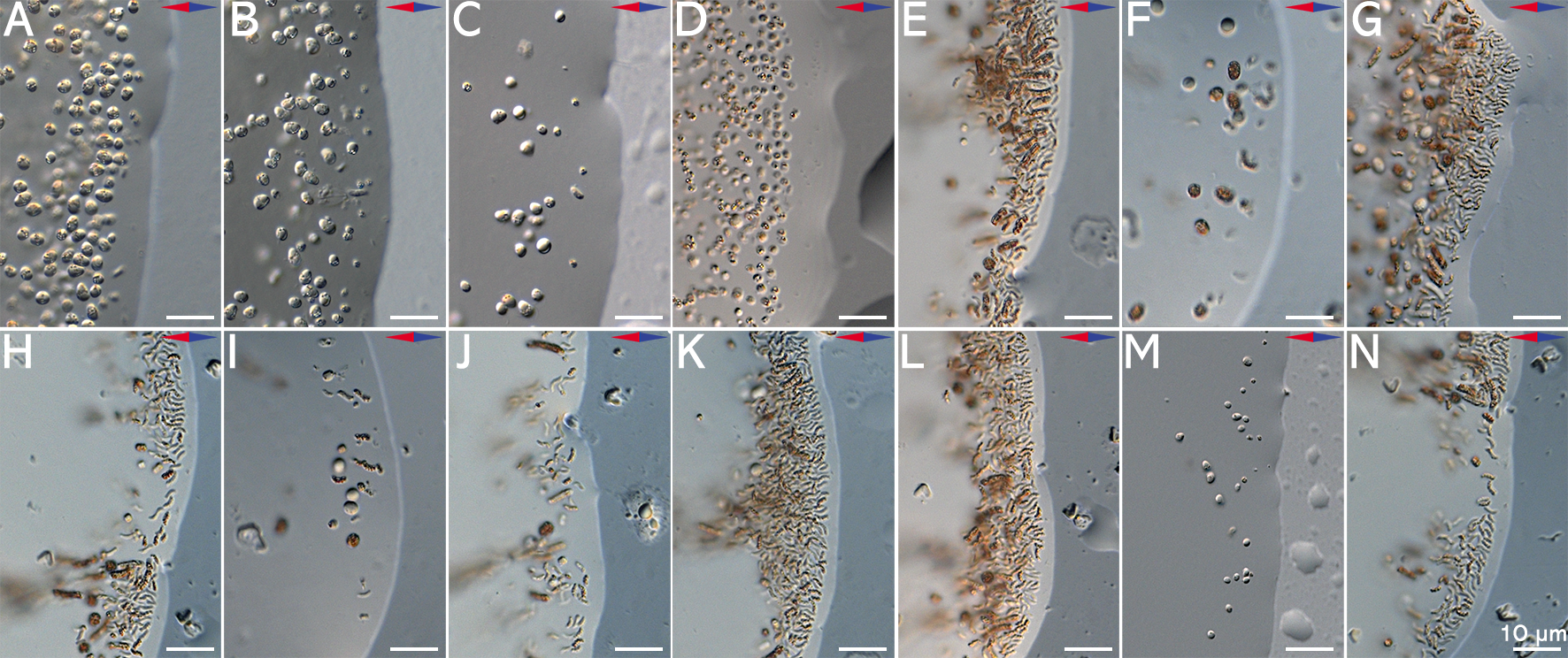
**

**Supplementary figure 2.** Magnetotactic response of magnetically enriched samples observed in DIC microscopy. Coccoid MTB are predominant within river main channel (A - P02; B - P05; C - P09; D - P13). Lakes and affluents harbor higher diversity of MTB morphotypes including rod-shaped bacteria, vibrioid cells, spirilla and ovoid cells (E - P01; F - P03; G - P04; H - P06; I - P07; J - P08 K - P10; L - P11; M - P12; N - P14). Compass, placed on the microscope's stage, represents the external magnetic field orientation. Magnetic north is represented in red whereas magnetic south is represented in blue. MTB, globally, exhibit south-seeking behavior, swimming antiparallel to the external magnetic field.

**Supplementary video 1.** Magnetotactic response of magnetically enriched samples from Araguaia floodplain’s lakes and affluents observed in DIC microscopy. Rods, spirilla, vibrioid and ovoid (both opaque and birefringent) cells are the majorly identified MTB morphotypes in lakes and affluents. Compass, placed on the microscope's stage, represents the external magnetic field orientation. Magnetic north is represented in red whereas magnetic south is represented in blue. MTB, globally, exhibit south-seeking behavior, swimming antiparallel to the external magnetic field.

**Supplementary video 2.** Magnetotactic response of magnetically enriched samples from Araguaia river main channel observed in DIC microscopy. Coccoid MTB, both large and small, are predominant in the river main channel. Compass, placed on the microscope's stage, represents the external magnetic field orientation. Magnetic north is represented in red whereas magnetic south is represented in blue. MTB, globally, exhibit south-seeking behavior, swimming antiparallel to the external magnetic field.

# 2. Supplementary materials and methods

## 2.1. Bioinformatic analyses

Bioinformatic analysis were performed using the mothur software version 1.44.3 [1]. Forward and reverse sequences were grouped in contigs. Primers and bar codes were removed previously by sequencing company. Sequences that contained ambiguities (N-base) or more than 8-mer homopolymers. Sequences that showed inconsistent size with amplicon were removed using command screen.seqs. Unique sequences were grouped together with unique.seqs command to reduce redundancy. Virtual PCR was performed in Silva database [2] with the command pcr.seqs. Sequences were aligned using align.seqs command. Non-informative columns and badly aligned sequences were removed. Sequences were trimmed to fully overlap and unique sequences were grouped once again. Sequences were pre-clustered with a difference threshold of 2 bp using pre.cluster command to artificially reduce errors. The chimeras were checked and removed using the chimera.vsearch command [3]. Virtual PCR were again performed for taxonomy classification using the Ribosomal Database Project [4]. Resulting reference file was used to classify our sequences using an 80% bootstrap threshold with classify.seqs command. Mitochondria, Chloroplast, Archaea and Eukaryotic contaminants were removed using remove.seqs command. OTUs were clustered with 3% of similarity cutoff with cluster.split and singletons were excluded with split.abund command. Randomized subsampling was performed to normalized samples by the smallest sequence file using sub.sample. Alpha- (*i.e.,* richness, diversity, dominance, evenness and rarefaction curve) and beta-diversity metrics (OTU relative abundance and distribution) were exported from software for later processing and plotting.

### 2.2. Alpha diversity

Alpha diversity metrics such as richness and diversity were obtained from the mothur output data as evenness and dominance indexes were obtained in Past 4.0 analysis. Both datasets were plotted in a boxplot format using GraphPad Prism software version 8.0. For bloxplot, chosen values for: (i) richness were the mean “chao” and upper and lower confidence values (“chao_lci” and “chao_hci”); (ii) diversity were the mean “shannon” and upper and lower confidence values (“shannon_lci” and “shannon_hci”); (iii) dominance were the mean “Dominance_D” and upper and lower confidence values; and (iv) evenness were the mean “Equitability_J” and upper and lower confidence values. A two-way ANOVA was selected for statistical inference between MC and LA samples.

A two-way ANOVA followed by Sidak's multiple comparisons post-test was chosen for statistical inference between relative abundance data. Statistical inference level, used in GraphPad Prism 8: (i) * for 0.05 > p > 0.0332; (ii) ** for 0.0332 > p > 0.0021; (iii) ** for 0.0021 > p > 0.0002; and (iv) **** for 0.0002 > p > 0.0001.

### 2.3. Beta diversity

The NMDS based on OTUs distribution was employed to determine the beta diversity of microbial communities in MC and LA samples. The groups were divided into MC and LA samples. The NMDS was calculated with the Bray-Curtis dissimilarity distance matrix and stress value for data accommodation using Past software version 4.0 [5]. One-way permutational multivariate analysis of variance (PERMANOVA) followed by Bonferroni correction for p-values was chosen to infer statistical differences between groups.

## 3. Supplementary results

## 3.1. OTU’s taxonomic assignment

A total of 183 different OTUs were sorted across the MC and LA samples, representing the clustering of microbial species into operational taxonomic units based on sequence similarity. This grouping highlights the shared and unique microbial diversity between the two samples, with species being classified into discrete OTUs to facilitate comparative ecological and phylogenetic analyses. At last, the three OTUs with the highest richness were identified as OTU0001, OTU0002, and OTU0003, and were taxonomically assigned to unclassified Betaproteobacteria, unclassified Bacteria, and unclassified Gammaproteobacteria, respectively. This classification highlights the dominance of these microbial groups in the samples, while their unclassified status underscores the presence of potentially novel or poorly characterized taxa within the microbial community or with no robust representatives related to their V3-V4 hypervariable region of the 16S rRNA gene.

**References**

1. Schloss, P. D., Westcott, S. L., Ryabin, T., Hall, J. R., Hartmann, M., Hollister, E. B., & Weber, C. F. (2009). Introducing mothur: open-source, platform-independent, community-supported software for describing and comparing microbial communities. Applied and environmental microbiology, 75(23), 7537-7541.
2. Quast, C., Pruesse, E., Yilmaz, P., Gerken, J., Schweer, T., Yarza, P., & Glöckner, F. O. (2012). The SILVA ribosomal RNA gene database project: improved data processing and web-based tools. Nucleic acids research, 41(D1), D590-D596.
3. Rognes, T., Flouri, T., Nichols, B., Quince, C., & Mahé, F. (2016). VSEARCH: a versatile open source tool for metagenomics. PeerJ, 4, e2584.
4. Cole, J. R., Wang, Q., Cardenas, E., Fish, J., Chai, B., Farris, R. J., & Tiedje, J. M. The Ribosomal Database Project: improved alignments and new tools for rRNA analysis. Nucleic Acids Research 2009, 37(suppl_1), D141-D145.
5. Hammer, Ø., Harper, D., & Ryan, P. (2001). PAST: paquete de programas de estadística paleontológica para enseñanza y análisis de datos. Palaeontol. Electrón, 4(1), 4.
